# The Prescribing and Physical Health Monitoring of Antipsychotic Medication for Patients With Dementia in a Community Treatment Team (CTT)

**DOI:** 10.1192/bjo.2024.531

**Published:** 2024-08-01

**Authors:** Nahrwan Alshamasi, Duncan Gray, Selina Clough

**Affiliations:** 1 Cumbria, Northumberland, Tyne, and Wear NHS Foundation Trust, Morpeth, United Kingdom; 2 Cumbria, Northumberland, Tyne, and Wear NHS Foundation Trust, Hexham, United Kingdom

## Abstract

**Aims:**

The aim of the audit was to assess compliance with prescribing standards for antipsychotics in patients with BPSD as outlined within NICE guidance and with trust policy, Physical Health Monitoring of Patients Prescribed Antipsychotics.

**Background:**

The Bannerjee report published in 2009 highlighted the problem of inappropriate use of antipsychotic medication in the treatment of patients with behavioral & psychological symptoms of dementia (BPSD).

When antipsychotic use is considered appropriate, good practice is imperative to minimize risk and ensure optimal outcomes for patients. This audit looked to assess whether the use of antipsychotics in patients within CTT with a diagnosis of dementia adhered to best practice standards as outlined by the Bannerjee report and NICE guideline. The audit looked to assess adherence to physical health monitoring requirements as per trust policy for patients prescribed antipsychotics. Currently, there is limited guidance around monitoring of antipsychotics for use in BPSD as they are not licensed in the longer term.

**Methods:**

A retrospective audit was undertaken for patients under the care of CTT between September 2020 and September 2021. 49 patients were prescribed an antipsychotic for BPSD.

**Results:**

Within the sample, 84% of patients were prescribed an antipsychotic at 12 months, 94% at 6 months and 98% at 3 months.

Compliance with the Audit standards showed: 82% of the patients had capacity assessed and documented prior to initiation of an antipsychotic.

98% of patients and/or carers had adverse effects of antipsychotics reviewed.

The risks and benefits of antipsychotics are discussed with the patient and/or carer(s) prior to antipsychotic initiation (94%). In 92% of patients, non-pharmacological interventions are tried prior to initiation of an antipsychotic. Clinical indications (target symptoms) are clearly documented (100%).

**Conclusion:**

Although good prescribing practice was demonstrated, there was an area of concern due to a lack of compliance with physical health monitoring requirements. Most patients were prescribed an antipsychotic for longer than the licensed treatment period.

Agreed Actions:

Discussion with all professionals to emphasise the necessity for effective communication and a documented care plan for antipsychotic monitoring and review.

Present and disseminate audit findings within locality groups and wider teams.

